# Personality, Stereotypy, and Responses to Crate Entry in Captive Giant Pandas

**DOI:** 10.3390/ani15243535

**Published:** 2025-12-08

**Authors:** Ming Li, Xueyang Fan, He Huang, Hao Zhang, Han Li, Xingna Zhao, Wenpei Peng, Hong Yin, Tao Deng, Kongju Wu, Mingxi Li, Kuixing Yang

**Affiliations:** 1 Chengdu Research Base of Giant Panda Breeding, Northern Suburb, Chengdu 610081, China; lm199305@163.com (M.L.); xf24@foxmail.com (X.F.); youngtree@163.com (H.H.); zr851123@126.com (H.Z.); leehanpda_7575@163.com (H.L.); dongguanzna@163.com (X.Z.); p412290914@outlook.com (W.P.); pandayh2015@163.com (H.Y.); dtlovexm@163.com (T.D.); wkjpanda@163.com (K.W.); lmxtr@hotmail.com (M.L.); 2 The Conservation of Endangered Wildlife Key Laboratory of Sichuan Province, Chengdu Research Base of Giant Panda Breeding, 1375 Panda Road, Chenghua District, Chengdu 610081, China

**Keywords:** giant panda, stereotypic behavior, personality, cortisol

## Abstract

Understanding how individual personality traits relate to stress and abnormal behaviors is essential for improving animal welfare and management. However, few studies have quantitatively examined these relationships in giant pandas. This study aimed to investigate how personality traits predict stereotypic behavior and physiological stress responses in captive giant pandas under a simulated transport context. Stereotypies are abnormal repetitive behaviors thought to reflect stress coping, and personality is increasingly recognized as a factor shaping animal welfare. We compared non-stereotypic (*n* = 16) and stereotypic (*n* = 16) giant pandas using keeper-based ratings, crate-entry training, and urinary cortisol. Intraclass correlation and principal component analyses identified two personality components, activity and timidity. Non-stereotypic pandas were significantly less timid (*p* = 0.005), whereas activity did not differ (*p* = 0.355). In crate-entry training, non-stereotypic pandas tended to enter faster (48.5 vs. 139.5 s; *p* = 0.074), and timidity was strongly correlated with latency (ρ = 0.74, *p* < 0.01). Censoring-aware analyses treating 300 s as right-censoring further supported this pattern (stratified Cox (Hazard Ratio) HR = 3.09, 95% CI 0.93–10.25; likelihood-ratio *p* = 0.052; log-normal accelerated failure-time model time ratio = 0.36, *p* = 0.054). Urinary cortisol showed no between-group difference after adjustment (geometric mean ratio = 0.93, 95% CI 0.57–1.49; *p* = 0.75), with consistent sensitivity analyses. These findings indicate that stereotypic pandas are more timid, and higher timidity predicts slower adjustment to a stress context, highlighting the value of personality assessment in management to mitigate stereotypies and improve welfare, while inconsistent cortisol results warrant further study.

## Simple Summary

To explore how personality relates to stress and abnormal behaviors in captive giant pandas, we compared 16 pandas showing stereotypic behaviors, such as pacing or rocking, with 16 non-stereotypic individuals. Using keeper ratings, two personality types emerged: activity and timidity. Stereotypic pandas were significantly more timid, and those scoring higher in timidity took longer to enter a transport crate during training, suggesting they adapted more slowly to stressful situations. Surprisingly, stress hormone levels in urine did not differ between groups. These findings show that personality influences welfare outcomes and can help improve care strategies for captive pandas.

## 1. Introduction

Personality (consistent individual differences in behavior) has been summarized into five broad dimensions—exploration–avoidance, shyness–boldness, sociability, activity, and aggressiveness—capturing major axes in the field [1]. Personality is commonly assessed via ratings by humans familiar with the animals and behavioral coding from ethograms under standardized tests or focal sampling [2]. Although terms such as “behavioral syndromes,” “coping styles,” and “temperament” are also used; however, these constructs converge on consistent individual differences in behavior [3]. Personality information can guide translocations, mate choice, breeding, and animal husbandry/management decisions [4].

From a management perspective, stereotypies in captivity remain a major concern: persistent stereotypic behavior can affect disease susceptibility, clinical injury, reproduction, and public education outcomes [5,6,7,8]. Determinants of stereotypies include intrinsic factors (e.g., life history, genetics) [9,10,11] and environmental factors (e.g., social grouping, suboptimal environments, management practices) [10,12,13,14,15]. Interventions have focused on positive reinforcement behavioral training [16], enrichment [17,18], social housing/grouping [19,20], and exhibit/environment optimization [21,22], yet longitudinal evaluations remain scarce.

Beyond environmental and management factors, individual personality itself can influence stereotypic behavior and adrenocortical activity. Compared with “confident” horses, those characterized as “aggressive,” “insecure,” or “irritable” exhibit more abnormal behaviors [23]. Shy chinchillas (*Chinchilla lanigera*) [24] and Asiatic lions (*Panthera leo persica*) [25] also display higher levels of stereotypy, supporting a link between personality and abnormal behavior.

In African lions, greater affiliative tendencies are associated with lower cortisol [26]; in African elephants, morning cortisol correlates positively with fearfulness and negatively with sociability and aggressiveness [27]; and in musk deer (*Moschus chrysogaster*), boldness is negatively related to fecal cortisol concentrations [28]. These findings suggest that individual differences in personality are related to adrenocortical activity. Moreover, stereotypic behavior itself may also covary with adrenocortical responses. Captive male alpine musk deer under social stress exhibit higher levels of both fecal cortisol and stereotypic behavior [29], and juvenile cynomolgus monkeys (*Macaca fascicularis*) housed in restrictive environments show increased stereotypies accompanied by elevated cortisol levels [30].

Cortisol, a widely used indicator of stress, reflects activation of the hypothalamic–pituitary–adrenal (HPA) axis, which complements the immediate sympathetic response to acute challenges [31]. Taken together, these studies indicate that personality differences may influence animals’ perception of stress, thereby producing consistent variation in behavioral and physiological outcomes.

Beyond welfare outcomes, personality also influences learning ability and training success in animals. Meta-analytic and experimental evidence shows that bolder or more exploratory individuals often acquire new tasks faster and exhibit greater behavioral flexibility, whereas shyer or more reactive animals tend to require longer to adjust to novel or stressful contexts [32,33]. Such personality–learning interactions may also shape how animals respond to routine management procedures such as crate-entry training. Few studies have simultaneously examined stereotypy, personality, and physiological stress indicators in giant pandas. The giant panda, a flagship species endemic to China, has been downlisted to *Vulnerable* [34], yet captive welfare remains under scrutiny. Giant panda research has largely focused on ecology and conservation [35], energetics and physiology [36], gut microbiota [37], reproduction [38], and behavioral rhythms in captivity [39]. By comparison, work that directly links personality to welfare remains relatively limited in this species. Existing findings indicate that personality can shape breeding outcomes and mate matching in captive pandas [38]. Welfare interventions have primarily centered on enrichment and selected management adjustments [40,41,42], yet an individual-targeted, personality-informed framework for husbandry is still underdeveloped. Moreover, few studies have jointly examined stereotypic behavior, personality traits, and physiological stress within a single analytical framework.

Against this backdrop, we compared personality characteristics, behavioral responses in a transport-like stress context, and urinary cortisol between captive pandas with and without stereotypies, and explored associations among these variables. Based on previous evidence linking personality, abnormal behavior, and stress physiology in mammals, we hypothesized that individual differences in personality would explain variation in behavioral and physiological responses to stress among pandas. Specifically, we predicted that (1) pandas with stereotypies would score higher on timidity than non-stereotypic individuals; (2) higher timidity would be associated with longer crate-entry latency (i.e., slower adjustment) in the stress context; and (3) pandas with stereotypies would exhibit higher urinary cortisol, with cortisol positively related to timidity.

## 2. Materials and Methods

### 2.1. Animals and Housing

From January to October 2024, we identified 16 healthy adult pandas displaying varying levels of stereotypy at the Chengdu Research Base of Giant Panda Breeding (CRBGPB, Sichuan, China) by combining focal observations with husbandry records and compiled an ethogram of stereotypies (Table 1). Husbandry logs were completed daily by area-assigned keepers and reviewed by supervisors, recording weather, intake, activity, feces, body mass, medication, enrichment, and all abnormal behaviors. All records were also entered into the institution’s digital husbandry management system, which provides a centralized data archive used for longitudinal tracking and verification. Husbandry logs were based on continuous observation during each keeper’s routine, and all occurrences of abnormal behaviors were recorded when they appeared. Focal observations were conducted by management staff to verify the presence or absence of stereotypy following standardized ethological procedures (see Table 1). These focal sessions used continuous recording for their full duration (typically 1–2 h) and were performed in both morning and afternoon periods. To ensure that abnormal behaviors were distinguished from context-appropriate responses, we applied standard criteria for stereotypy following criteria and previous studies [43,44]: behaviors had to be repetitive, invariant in form, occur in the absence of an immediate functional goal or acute disturbance, and be observed across multiple days. An individual was classified as stereotypic if abnormal repetitive behaviors were recorded in both logs and focal observations on ≥3 days or ≥2 independent sessions; otherwise, the panda was considered non-stereotypic. Brief movements clearly directed toward withdrawal from a momentary disturbance were not counted as stereotypy. For example, “walk backward” was classified as stereotypic only when pandas repeatedly walked backward along a fixed route under calm conditions, and not when taking a few backward steps in response to an acute stimulus. In this study, stereotypy was treated as a qualitative grouping criterion rather than a continuous behavioral variable; pandas showing repetitive stereotypic behaviors were classified as the “stereotypic group”, and those without such behaviors as the “non-stereotypic group”. Then, 16 stereotypic adults formed the stereotypic group based on the husbandry reports; 16 non-stereotypic adults were randomly selected as the comparison group based on constraints on feasibility and approximate balance by husbandry area and sex (total *n* = 32; Table 2). The sample comprised 17 males and 15 females (mean age ± SD: 13.78 ± 6.53 years). Except for the two mothers housed with their cubs, pandas were singly housed to facilitate observation. All individuals were born at the Chengdu Research Base of Giant Panda Breeding and had experienced routine transport at least once as part of standard husbandry procedures. None had been transported or received transport-related training within at least six months before the study, ensuring that no animal had recent exposure that could affect crate-entry responses.

Each panda had an indoor den (with a water bowl and bed) and an outdoor yard (with climbing structures, a pool, a simulated stream, and trees/shrubs). The indoor dens and outdoor yards were connected by a guillotine door. When a panda approached the doorway, keepers opened the door to allow voluntary access between the two areas. Giant pandas prefer cool temperatures and typically remain most active within a moderate thermal range. Liu et al. [45] reported that the suitable temperature range for giant pandas is cool. During cool weather, pandas were released outdoors at around 08:30 and returned indoors by approximately 17:30. When ambient temperatures exceeded 26 °C, keepers gradually guided pandas back indoors to ensure thermal comfort. All individuals followed the same daily schedule. Keepers provided continuous dietary care, patrols, and monitoring, and all pandas received daily enrichment as part of standard husbandry practice.

### 2.2. Ratings (Keeper-Based Assessment)

Keeper ratings are a reliable approach widely used in zoos to evaluate personality [46]. Ratings distill observers’ experience of animals across extended periods and situations, thereby integrating the core requirement of repeatability into a composite variable. Building on prior panda research [47,48], we designed a 19-item, five-point Likert survey (1 = not at all to 5 = very much). Trait definitions were provided to raters (Table 3).

To improve accuracy, we recruited four stable and experienced keepers or management staff as raters in each husbandry area, all with at least two years of professional experience. One supervisor oversaw two areas (Areas 2 and 3), yielding a total of 19 raters. Before completing the survey, all raters reviewed and discussed the definitions of all 19 items to ensure a consistent understanding of each trait and could identify representative behavioral examples corresponding to each trait (without reference to specific individuals). This preparatory session served as a formal training to standardize rating criteria, ensuring that rater consistency was achieved through explicit instruction rather than experience alone.

The Chengdu Research Base of Giant Panda Breeding (CRBGPB) is divided into five areas; keepers rotate across all areas, which can blur impressions of particular individuals. To improve rating accuracy for this study, however, a fixed team of raters was designated for each area to ensure consistency and familiarity with the pandas being evaluated. Ratings were collected between November 2024 and April 2025, allowing raters to strengthen familiarity before completing surveys. Each panda was rated independently by four raters from its respective area, and we received 128 individually completed questionnaires. Raters completed independent evaluations without discussion. All raters demonstrated adequate consistency and none were disqualified. Inter-rater reliability was subsequently evaluated using intraclass correlation coefficients (ICC [3, 1] and ICC [3, K]); traits with inadequate reliability were excluded as detailed in Section 2.5.

### 2.3. Crate-Entry Training (Transport Simulation)

Transport is common stressor for captive species; crating is the first step for pandas. During crating, some individuals show marked stress (e.g., vocalizing, running, climbing bars, avoidance, frequent defecation, stereotypies), whereas others remain calmer. Individuals that enter transport crates voluntarily and calmly typically show better condition. Positive reinforcement training has been used in other taxa to mitigate transport-related impacts [49,50,51]. Accordingly, crate-entry training was incorporated into routine training to enhance adaptability. Between May and June 2025, 28 pandas participated; four did not due to maternal care or management.

Because pandas prefer cool temperatures, as noted above [45], training sessions were scheduled in the morning (~11:00) before indoor temperatures increased. During the summer, pandas were brought indoors as temperatures rose to maintain thermal comfort and minimize stress during crate-entry procedures. Training began around 11:00 when pandas were not fully satiated. After installing a transport crate in the indoor den, bamboo shoots and apple were placed at the far end as food reinforcement. The type and ratio of reinforcement (two small bamboo shoots and one apple slice per trial) were kept consistent across all sessions. Timing (crate-entry latency, seconds) started when the crate door opened and stopped when the panda fully entered and obtained the food. A 5 min limit was set; if food was not obtained, keepers calmly intervened and timing stopped. Latency was always recorded before any soothing occurred. When pandas hesitated or showed clear avoidance behaviors, keepers first provided gentle verbal encouragement and briefly stepped back from the crate door to reduce perceived pressure. In occasional cases, a single additional piece of apple—identical to the standard food reward—was offered to help re-establish approach behavior. No honey or other food types were used in the trials analyzed in this study. This brief keeper-led calming process, typically lasting less than 1 min and applied under the same criteria for all individuals, was defined as “soothing.” Behavioral training (part of routine husbandry but not part of this study’s experimental measurements) proceeded only after latency recording was completed.

All pandas followed the same standardized procedure. To avoid habituation to the simulated transport context, only the latency from the first training session was used for analysis. Accordingly, each panda contributed a single latency measurement, taken from its first standardized crate-entry training session. Crate-entry performance was not included in the personality assessment; instead, crate-entry latency (seconds) and urinary cortisol (below) indexed behavioral and physiological responses to this stress context.

### 2.4. Measurement of Urinary Cortisol

Urinary cortisol levels were expressed as cortisol normalized to creatinine (ng cortisol per mg creatinine). This normalization was applied because spot urine samples can vary substantially in concentration depending on each animal’s hydration status and voiding volume. Adjusting for creatinine thus controls for urine dilution and provides a more reliable index of adrenal activity. The standardized cortisol/creatinine ratio was therefore used in all subsequent analyses. Cortisol concentrations were measured using an enzyme immunoassay previously used in studies of this species, and creatinine concentrations were determined by the colorimetric Jaffé reaction as described below.

Urine was collected on the same day after the training session under quiet conditions, defined as temporarily suspending visitor access to the viewing area, closing the service corridor adjacent to the enclosure, minimizing staff movement and routine noise, and ensuring doors to neighboring dens remained closed during sampling. To further minimize external disturbances, no additional husbandry operations or construction activities were conducted during this period, and pandas were provided with their normal post-training food to maintain routine conditions. Training started at ~11:00. To control for diurnal variation in glucocorticoids, all urine samples were collected at ~14:00, yielding an interval of approximately 3 h between training and sampling. A single post-training urine sample was collected from each panda, resulting in 14 samples per group and 28 samples in total. Both stereotypic and non-stereotypic groups followed identical procedures under these standardized conditions, and no separate non-training baseline samples were collected, as the aim was to compare responses between groups under matched experimental conditions.

Although some studies have reported that urinary glucocorticoid metabolites peak many hours after acute stress, more recent evidence suggests that this delay is highly species-dependent. Verspeek et al. [52] found that in bonobos (*Pan paniscus*), urinary and salivary cortisol peaked about 160 min after a psychological stressor, indicating that measurable endocrine responses can occur within only a few hours. To date, no validation study has determined the exact excretion lag for giant pandas. However, Wang et al. [53] collected urine within 3 h after stress-related behavioral events in captive adult pandas and successfully detected cortisol elevation. Guided by these findings, we adopted a fixed 3 h post-training window (training 11:00, sampling 14:00) to minimize circadian effects and ensure procedural consistency across individuals.

Keepers visually confirmed urination before sample collection. Immediately after urination, the panda was calmly guided to an adjacent pen, allowing staff to collect the fresh sample from the enclosure floor. Urine was drawn within 1–2 min using sterile disposable syringes directly from a smooth epoxy-coated concrete substrate, which prevented absorption and minimized the risk of contamination. The sample (≈2 mL) was transferred into labeled cryotubes, centrifuged (3000 rpm, 10 min) (Heraeus Instruments, Hanau, Germany), and stored at −20 °C until cortisol and creatinine assays were performed.

Creatinine was quantified colorimetrically after 1:20 dilution. Briefly, 50 μL of standards and diluted urine were added to microplate wells, followed by 50 μL distilled water, 50 μL 0.75 mol/L NaOH, and 50 μL 0.04 mol/L picric acid, and incubated 20 min at room temperature (18–25 °C). Absorbance was read at 490 nm (Thermo Fisher Scientific, Waltham, MA, USA), and concentrations were calculated from standard curves. Samples with creatinine < 0.1 mg/mL were considered over-diluted/contaminated and excluded; all samples met criteria.

For cortisol, enzyme-linked immunosorbent assays (ELISAs) were performed on plates coated with anti-rabbit IgG. ELISA buffer, standards, diluted urine, cortisol–HRP, and antibody R4866 were added and incubated 1 h at 37 °C. Plates were washed, chromogenic solutions were added and incubated 30 min at 37 °C, and reactions were stopped with 4 mol/L H_2_SO_4_ (50 μL/well). Absorbance was read at 450 nm, and concentrations were obtained from standard curves. Cortisol values (ng/mL) were normalized to creatinine (mg/mL) and expressed as ng/mg Cr [53,54].

### 2.5. Data Analysis

All analyses were run in IBM SPSS Statistics 26 (IBM Corp., Armonk, NY, USA); time-to-event analyses were conducted in R (R Foundation for Statistical Computing, Vienna, Austria, version 4.4; packages survival, survminer); *p* < 0.05 denoted significance.

#### 2.5.1. Inter-Rater Reliability

In this study, “traits” refer to the 19 personality adjectives listed in Table 3, and ICCs were computed for each trait within each husbandry area to assess agreement among the four raters per area. Intraclass correlation coefficients (ICC) are widely used statistical approach for quantifying inter-rater consistency in behavioral and psychological research. Each husbandry area at the CRBGPB has its own fixed team of keepers and managers, who exclusively evaluated pandas within their assigned area. Because raters across areas did not assess the same individuals, inter-rater reliability was assessed separately within each area to ensure that agreement reflected ratings of the same animals by familiar observers.

Each rater had received prior orientation and reviewed standardized trait definitions before completing the surveys (see Section 2.2), ensuring consistent understanding of each behavioral adjective and its representative examples. We used two ICC to quantify inter-rater reliability [55]: ICC [3, 1] for single raters and ICC [3, K] for the mean of *K* raters [56]. These indices specifically measure consistency among raters’ scores for the same trait, rather than the internal reliability of the questionnaire itself. The two-way mixed-effects ICC model (type 3) was chosen because all raters were fixed and rated the same set of individuals. Traits with ICC < 0.5 were excluded from further analyses. Thus, personality scores were not determined by averaging conflicting ratings; instead, only traits with adequate inter-rater consistency were retained, ensuring that disagreements among raters did not contribute to the derived personality components. If a trait was excluded in one husbandry area, it was excluded across all areas to retain a consistent trait set for Principal component analysis (PCA). No raters were excluded, as the goal was to identify reliable traits rather than evaluate the raters themselves. This approach ensured that only traits showing consistent scoring patterns across all areas were retained for subsequent analyses. All ICCs were computed in IBM SPSS Statistics using the Reliability Analysis function. An ICC value of 0.5 was considered the minimum acceptable threshold for moderate agreement, following conventions in behavioral research.

#### 2.5.2. Principal Component Analysis (PCA)

Averaged trait scores per panda were subjected to PCA with varimax rotation. Sampling adequacy was evaluated by the Kaiser–Meyer–Olkin (KMO) statistic and Bartlett’s test of sphericity. Based on scree plots and eigenvalues > 1, traits were assigned to components on which they loaded highest. Internal consistency was evaluated with Cronbach’s α; α ≥ 0.7 indicated acceptable consistency. Component scores were computed as the mean of traits loading on each extracted component. In subsequent analyses, the two resulting components were interpreted as representing general activity and timidity based on their highest loading traits (see Section 3).

#### 2.5.3. Group Comparisons and Correlations

Shapiro–Wilk and Levene’s tests examined normality and homoscedasticity. Depending on distributions and variances, independent samples *t*-tests or Mann–Whitney U tests compared non-stereotypic vs. stereotypic groups; data are presented as median (25th–75th percentiles).

To examine predictors of stereotypic status, we fitted a logistic regression model with stereotypic group membership (yes/no) as the dependent variable and Timidity and Activity as predictors. To assess how personality and group membership jointly related to behavioral and physiological responses, we further fitted multiple linear regressions with log-transformed crate-entry latency and log-transformed urinary cortisol as dependent variables, and Timidity, Activity, and stereotypic status as predictors.

In addition, Spearman’s rank correlations were computed to provide an exploratory description of bivariate associations among the behavioral and physiological variables (Activity, Timidity, crate-entry latency, and urinary cortisol). Given the small number of planned tests and their basis in a priori hypotheses, we did not apply formal multiple-comparison corrections but interpret *p*-values cautiously.

#### 2.5.4. Time-to-Entry (Crate-Entry Latency; Censoring-Aware Analysis)

Because a 5 min cap was imposed, trials not completed within 300 s were treated as right-censored. Group differences were visualized with Kaplan–Meier curves and tested with log-rank tests. We then fitted Cox proportional-hazards models with Group as the main predictor and assessed the proportional-hazards assumption using Schoenfeld residuals. Given five husbandry areas that may differ in baseline entry rates, we additionally fitted stratified Cox models with Area (different sections that are keeping pandas) as the stratification factor (area-specific baseline hazards; common group effect). As a complementary specification that models time scaling directly, we fitted a log-normal accelerated failure-time (AFT) model and reported the time ratio (TR; <1 denotes faster completion). A Mann–Whitney U test that treats 300 s as observed values is reported as a non-censoring sensitivity analysis.

## 3. Results

### 3.1. Inter-Rater Reliabilities

Six of the nineteen traits met the reliability criterion in all five areas—Active, Alert, Adverse reactions to noise, Forage, Sensitive, and Utilization of enrichment—and were retained for subsequent analyses. Within areas, ICC [3, 1] values ranged as follows: Area 1, 0.502 (Active) to 0.667 (Forage); Area 2, 0.526 (Utilization of enrichment) to 0.894 (Active); Area 3, 0.500 (Active) to 0.756 (Forage); Area 4, 0.513 (Alert) to 0.778 (Active); Area 5, 0.524 (Alert) to 0.667 (Forage). As expected, ICC [3, K] exceeded ICC [3, 1] throughout, and reliabilities fell within accepted ranges.

### 3.2. Principal Components Analyses

**Figure 1:**
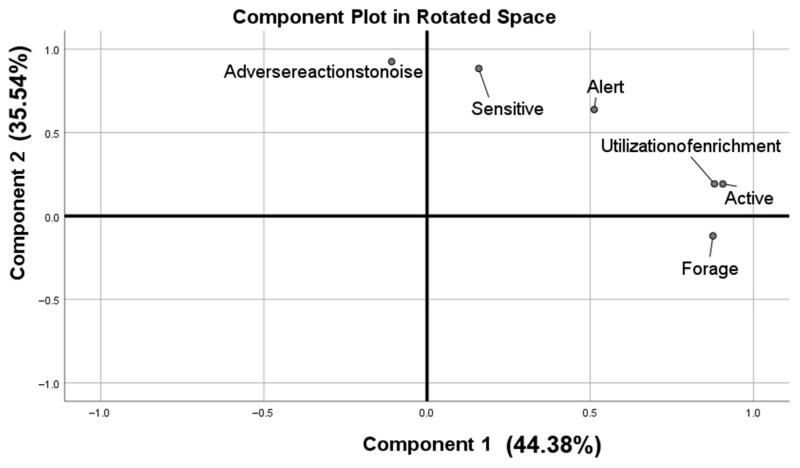
Personality in relation to components of the Principal Component Analysis. The first component was related to Active, Utilization of enrichment, Forage, and the second component was related to Adverse reactions to noise, Sensitive, Alert.

Sampling adequacy was supported by a KMO = 0.713 and a significant Bartlett’s test (*p* < 0.01). Inspection of the scree plot and the >1 eigenvalue rule indicated two principal components (PCs) that together explained 79.918% of the total variance. After varimax rotation, traits were assigned to the component on which they loaded highest (Figure 1; Table 4). Active, Forage, and Utilization of enrichment captured overall activity and defined PC1 (Activity); Alert, Adverse reactions to noise, and Sensitive reflected responses to risk or disturbance and defined PC2 (timidity).

### 3.3. Group Differences (Non-Stereotypic vs. Stereotypic)

**Figure 2:**
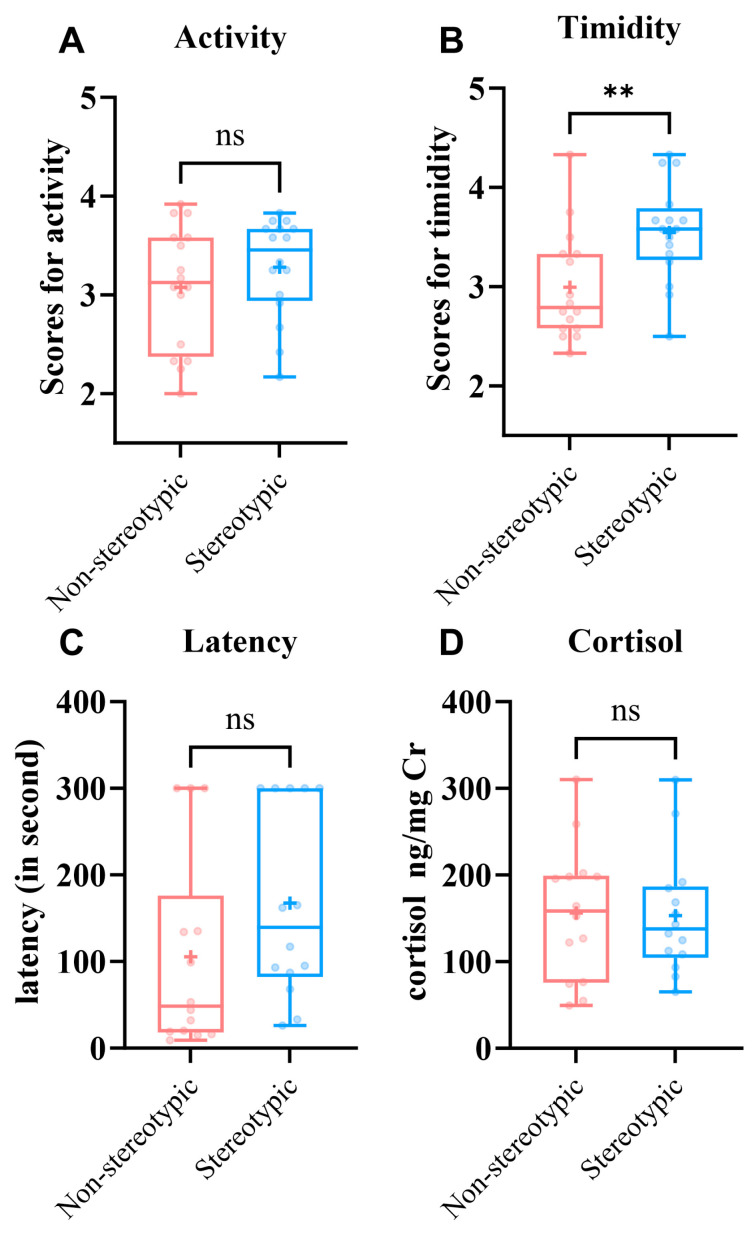
Between-group differences in personality and responses to a transport context in giant pandas. (**A**) Activity. Differences in Activity scores between the non-stereotypic (*n* = 16) and stereotypic (*n* = 16) groups. (**B**) Timidity. Differences in timidity scores between the non-stereotypic (*n* = 16) and stereotypic (*n* = 16) groups. (**C**) Differences in latency. scores between the non-stereotypic (*n* = 14) and stereotypic (*n* = 14) individuals. (**D**) Cortisol. Urinary cortisol (ng/mg Cr) in non-stereotypic (*n* = 14) and stereotypic (*n* = 14) individuals. (ns, not significant; “+”, mean; ** *p* < 0.01).

To compare personality and responses to a stress context, we contrasted personality scores, crate entry latency, and urinary cortisol between the non-stereotypic and stereotypic groups (Figure 2).

- Activity (Figure 2A), there was no significant difference between groups—non-stereotypic: 3.125 (2.46–3.58) vs. stereotypic: 3.458 (2.855–3.688); Z = −0.926, *p* = 0.355.
- Timidity (Figure 2B), the non-stereotypic group scored lower—2.99 ± 0.55 vs. 3.55 ± 0.49—and the difference was statistically significant (*t* = −3.003, *p* = 0.005).
- Crate entry training (Figure 2C), the non-stereotypic group entered the transport crate faster, with a latency of 48.5 s (18.25–176.25) vs. 139.5 s (82.25–300.00) in the stereotypic group; the difference was not statistically significant (Z = −1.790, *p* = 0.074), although non-stereotypic pandas tended to enter faster on average.

Treating 300 s as right-censoring, non-stereotypic pandas tended to enter faster (stratified Cox HR = 3.09, 95% CI 0.93–10.25; LR *p* = 0.052). A log-normal AFT model was consistent (TR = 0.36; *p* = 0.054); KM curves showed the same direction (log-rank *p* = 0.20). Urinary cortisol did not differ between groups—non-stereotypic: 156.05 ± 77.58 ng/mg Cr vs. stereotypic: 153.13 ± 69.23 ng/mg Cr (*t* = 0.105, *p* = 0.917; Figure 2D). Overall, stereotypic pandas showed higher timidity and a tendency toward longer crate-entry latencies, although this difference was not statistically significant and may reflect variability among a few individuals reaching the 300 s limit.

### 3.4. Spearman’s Rank Correlations

**Figure 3:**
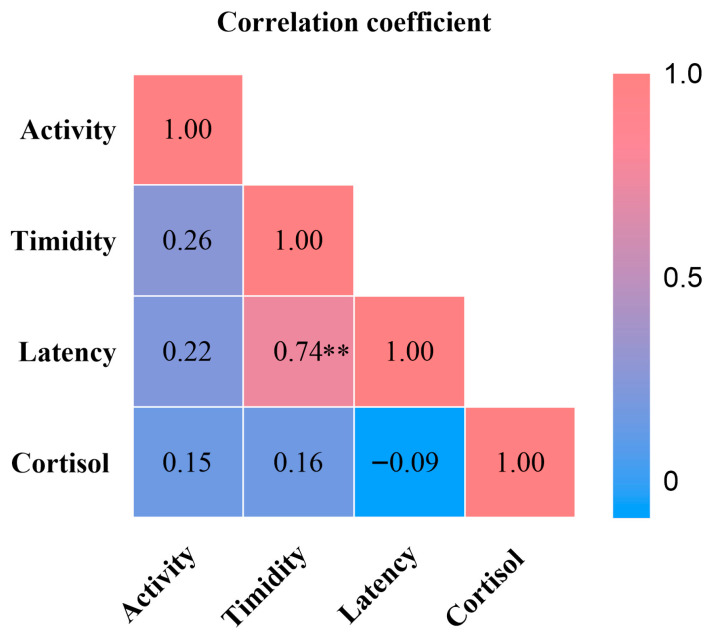
Heatmap of Spearman’s Rank Correlation Coefficients. ** *p* < 0.01.

We examined associations between personality traits and crate entry latency and urinary cortisol (Figure 3). Timidity was strongly and positively associated with crate entry latency (ρ = 0.74, *p* < 0.01). Urinary cortisol showed no significant association with activity or timidity. These results indicate that individuals with higher timidity require more time to cope with or adapt to the stress context.

### 3.5. Regression Analyses

Logistic regression with stereotypic status as the outcome showed that Timidity was a significant positive predictor of belonging to the stereotypic group (odds ratio = 7.75, 95% CI 1.63–62.19, *p* = 0.023), whereas Activity was not significant (*p* = 0.72). Thus, higher Timidity was associated with an increased likelihood of showing stereotypic behavior when controlling for Activity. In a multiple regression with log-transformed crate-entry latency as the dependent variable, Timidity remained a strong positive predictor (β = 1.32 ± 0.30 SE, *t* = 4.48, *p* < 0.001), whereas Activity and stereotypic status were not significant (both *p* > 0.45). This indicates that individual differences in Timidity, rather than group label per se, accounted for variation in latency. In contrast, a multiple regression on log-transformed urinary cortisol revealed no significant effects of Timidity, Activity, or stereotypic status (all *p* > 0.20), consistent with the absence of group differences in cortisol reported above.

## 4. Discussion

Using keeper-based ratings, we derived two reliable personality components—activity and timidity—in captive pandas. Although a conservative reliability threshold resulted in many traits being excluded, similar patterns have been reported in other species [26,57]. The emergence of activity and timidity aligns with previous panda personality studies, suggesting these dimensions are readily identifiable in this species. Keeper-derived personality assessments are widely adopted in zoo-animal research, particularly when combined with formal measures of inter-rater reliability [2,46]. In giant pandas, studies using this framework have shown that reliably rated traits can predict meaningful outcomes such as reproductive behavior and mate compatibility [47,48].

Consistent with this established practice, our study used standardized trait definitions, trained raters, and area-specific ICC calculations to ensure trait-level reliability prior to PCA extraction. Only traits meeting the reliability criterion across all areas were retained. Together, this procedure ensured that the resulting Activity and Timidity components reflect stable behavioral differences rather than artefacts of subjective variation. Nevertheless, the ICC-based screening procedure also has methodological limitations. Because ICCs were calculated separately within each husbandry area using a relatively small, fixed panel of raters, the resulting estimates may be sensitive to local husbandry culture and rater composition. In addition, by excluding all traits that fell below the a priori ICC threshold in any area, we may have discarded biologically meaningful variation and constrained the dimensional structure of personality. Future work could mitigate these issues by increasing the number and diversity of raters, incorporating formal video-based calibration sessions, and re-evaluating reliability thresholds in larger or multi-site samples.

While activity did not differ between groups, non-stereotypic pandas were less timid than stereotypic pandas. Cross-species work also links greater shyness/fearfulness to higher levels of stereotypy, as in chinchillas [24] and Asiatic lions [25]. Early work on coping styles distinguished “proactive” and “reactive” profiles [58]; proactive styles have been associated with stereotypies—for example, African grey parrots (*Psittacus erithacus*) with more proactive coping show greater feather damage [59], and stereotypic rats display more proactive responses (e.g., escape) in open-field tests [60]. Conceptually, coping style overlaps with personality but is narrower in scope; proactive-style behavioral features have been subsumed under Timidity because such individuals more readily exhibit flight/heightened reactivity [61]. In addition, “neuroticism” has been associated with increased risk of stereotypic behavior [62,63]. Individuals high in neuroticism tend to be emotional, anxious, tense, and sensitive, thereby heightening the perception of stressors [64,65]. Thus, timidity, neuroticism, and the proactive profile appear to overlap substantially in definition. Cross-species studies and the present findings converge to suggest that the link between Timidity and stereotypy may be widespread across taxa. In our dataset, this relationship remained robust in a logistic regression model, where higher Timidity significantly increased the odds of belonging to the stereotypic group after controlling for Activity. According to Réale et al.’s definition of the shyness–boldness dimension [1], stereotypic individuals, by virtue of higher timidity, may adapt more poorly and experience higher stress levels under stress contexts.

In practice, we observed clear individual differences during crate-entry training. More tense individuals displayed obvious stress reactions (e.g., roaring, running, climbing the bars, avoidance, frequent defecation, and stereotypies). Although we did not quantify these behaviors, they were objectively present. To safeguard welfare, we did not require pandas to remain in the stress context for a fixed duration to obtain behavioral data; instead, within a 5 min window we recorded the latency to voluntarily enter the transport crate to obtain food.

The lack of a significant group difference in crate-entry latency should be interpreted in light of methodological constraints. Because several pandas did not enter within the 300 s cap, the data included right-censored observations and a restricted range for estimating between-group differences. These features, combined with the modest sample size typical of great-ape and large-carnivore studies, reduce statistical power and increase uncertainty around effect estimates. Nevertheless, the consistent directional trend across non-parametric and survival-analytic approaches suggests that stereotypic individuals may take longer to initiate entry, but the present study was underpowered to detect this reliably.

Cross-species studies on shy or timid personalities have suggested that latency is an important indicator—more timid individuals generally require longer to adapt to stressful contexts [28,66,67,68]. However, no study to date has directly examined the relationship between timidity and crate-entry latency in giant pandas. In our dataset, timidity scores were strongly and positively correlated with latency. A multiple regression analysis with log-transformed latency further showed that Timidity remained a strong predictor even when Activity and stereotypic status were included in the model, indicating that individual differences in Timidity, rather than group label per se, drove variation in crate-entry latency. Taken together, timidity can help predict animals’ adaptability to stress contexts, and a complementary log-normal AFT model likewise suggested shorter completion times in non-stereotypic pandas (time ratio = 0.36; *p* = 0.054). This pattern is also consistent with personality–learning links, where more timid or reactive individuals often show delayed learning or slower adaptation to novel training contexts [32,33]. These findings suggest that timidity can serve as a behavioral predictor of adaptability to stress contexts.

Previous research indicates that shyer, more fearful individuals—when adapting poorly—tend to show elevated cortisol. For example, in musk deer, boldness is negatively correlated with fecal cortisol metabolites [28]; shy squirrels exhibit greater vigilance toward novel objects and the highest fecal cortisol concentrations [69]; bolder dogs show better skills for coping with stress contexts and lower disease susceptibility [5]. Based on this evidence, we hypothesized that the stereotypic pandas might experience greater stress due to higher timidity, leading to higher cortisol. However, our results showed no significant between-group difference in cortisol, nor significant correlations of cortisol with personality component scores or latency, contrary to our expectation. Similarly, in rainbow trout (*Oncorhynchus mykiss*), bolder fish spend less time in novel-object tests, yet no direct association has been found between boldness and stress reactivity or gene expression [70].

To interpret these findings critically, several methodological considerations should be noted. All urine samples in this study were collected at approximately 14:00 h under quiet conditions to reduce diel variation in cortisol secretion, which exhibits marked diurnal and seasonal patterns in giant pandas [71]. However, no non-training baseline samples were obtained, because our aim was to compare responses between groups under matched procedures rather than to establish absolute baseline levels. This design, while minimizing inter-individual variability, limits our ability to distinguish whether cortisol elevations reflected acute stress induced by the crate-entry session or longer-term (chronic) differences.

Previous studies on giant pandas have adopted different sampling contexts. Yuan et al. [54] compared urine cortisol between socially housed and solitary sub-adult pandas and reported behavioral improvements but no significant difference in cortisol levels, illustrating the difficulty of detecting physiological contrasts even when behavior clearly differs. In contrast, Wang et al. [53] found that abnormal mating behavior in adult pandas was associated with elevated urinary cortisol, demonstrating that cortisol can reflect behavioral stress when sampling frequency and behavioral context are well aligned. These contrasting outcomes highlight that the interpretability of cortisol depends strongly on study design, sampling timing, and the type of stressor.

Overall, associations between personality traits and cortisol levels remain inconsistent across studies [5,28,67,68,72]. Under acute stress, activation of the sympathetic system triggers immediate physiological responses (e.g., increased heart rate, blood pressure, and respiration), whereas under chronic stress, sustained activation of the HPA axis can reveal adverse glucocorticoid effects [31]. Differences in stressors may lead to divergent outcomes. In addition, organismal response mechanisms incorporate genetic variation and the influence of life-history experience [73], underscoring the complexity of behavior–physiology coupling.

Our study had limitations. For animal welfare and husbandry reasons we avoided coercive exposure and novelty tests. Urine samples were collected shortly after the first crate-entry training session under calm conditions to minimize extraneous disturbances that could confound cortisol secretion. Compared with plasma cortisol, urinary cortisol represents the hormone’s accumulation over time and thus shows a temporal delay relative to acute stress peaks. Therefore, the samples likely reflected basal or recovery levels rather than immediate responses during the stress event. Moreover, there is currently no established theoretical basis for the time interval between acute stress and measurable changes in urinary cortisol in giant pandas. During crate-entry simulations, a 5 min limit and keeper soothing may have attenuated group contrasts and obscured correlations. Censoring at the cap also reduces power for the log-rank test; stratified Cox/AFT recovered the same direction of effect but intervals remained wide. It is also possible that cortisol measured under calm conditions underestimated transient stress responses that occurred during crate-entry training. Furthermore, the single post-session sampling without repeated baseline or in-session collections limited our ability to capture the full cortisol response curve, and the short observation window may have missed transient peaks altogether. In addition, although the soothing procedure was applied according to identical criteria across individuals and did not influence latency measurements—because latency was always recorded before any soothing occurred—we did not systematically quantify the frequency or duration of soothing. This unmeasured variation may have partially attenuated between-group differences in urinary cortisol, especially if brief calming reduced acute agitation in some individuals. We now recognize this as a methodological limitation of the current design.

Given the inherent variability of glucocorticoid measures, future longitudinal research should adopt repeated endocrine sampling and integrate complementary physiological and behavioral metrics—such as heart rate, respiratory patterns, and finer-grained behavioral coding—within an allostatic-load framework. Such multimodal approaches across the full transport procedure would clarify the temporal dynamics between stress responses and subsequent elevations in urinary or fecal cortisol, providing a more precise understanding of individual coping and stress adaptation. In practice, shy or highly timid pandas may benefit from graded desensitization and counter-conditioning programs, involving short and frequent training sessions, predictable routines, and individualized reinforcement to reduce stress and improve adaptability during crate-entry procedures.

## 5. Conclusions

Stereotypic and non-stereotypic giant pandas differed in personality and behavioral responses to a simulated stressor. Higher timidity in stereotypic individuals predicted longer crate-entry latency, indicating slower behavioral adjustment. Urinary cortisol concentrations, reflecting adrenocortical activity, did not differ significantly between groups and showed no correlation with personality dimensions. These findings suggest that while stereotypic pandas display heightened behavioral reactivity, their physiological stress response, as indexed by urinary cortisol, may not differ detectably under the current sampling conditions. Further research incorporating extended sampling windows or additional physiological indicators is warranted.

## Figures and Tables

**Table 1:** Ethogram of stereotypies in giant panda.

Stereotypic Behaviors	Definition
Stereotypic Pace	Back and forth or perimeter travels in a repetitive, sustained, stereotyped pattern.
Pirouette	Stands on hind legs and spins at least 90 degrees.
Head Toss	Abruptly lifts head upward and/or to the side in a swinging movement. Often occurs during pacing (especially when turning).
Rock	Shifts weight from side to side, but remains stationary. Often occurs in anticipation of feeding.
Roll	Frequently rolls its body while walking (especially when turning).
Walk Backward	Repetitive backward walking along a fixed route in calm conditions.
Suck	Sucks on parts of its own body (e.g., paws, chest), or the body of other individuals. This behavior is mainly seen in cubs.

**Table 2:** The information of giant pandas.

Area	Animal ID	Sex	Age	Stereotypic Behaviors
1	1	M	18	/
	2	F	18	/
	3	M	8	Rock
	4	F	13	/
	5	M	7	/
	6	F	9	Pirouette
	7	M	17	Stereotypic Pace, Roll
	8	M	7	Stereotypic Pace
2	9	F	10	Stereotypic Pace, Head Toss
	10	F	10	Stereotypic Pace, Head Toss
	11	M	7	Walk Backward
	12	M	23	/
	13	F	8	/
	14	M	19	Stereotypic Pace, Head Toss
	15	M	23	/
	16	F	17	Stereotypic Pace, Head Toss
	17	M	8	/
	18	F	20	/
	19	M	24	/
	20	F	25	/
3	21	M	9	/
	22	F	8	/
	23	F	21	Stereotypic Pace, Head Toss
	24	F	21	Stereotypic Pace, Head Toss
	25	M	6	/
4	26	M	5	Pirouette
	27	M	7	Stereotypic Pace, Head Toss
	28	F	12	Stereotypic Pace, Head Toss
	29	F	25	Rock
	30	M	9	Rock
5	31	F	11	/
	32	M	16	/

“/” indicates no stereotypic behavior recorded.

**Table 3:** Definitions of traits used in the keeper assessment survey.

Trait	Definition
Curious/Explorative	Readily approaches and explores changes in the environment
Adaptable	Adaptability to new environments.
Innovative	Solves problems and creates new behaviors (e.g., using new enrichments or tools).
Smart	Learns quickly to associate certain events and seems to remember for a long time.
Aggressive to people	Reacts in a hostile way or attempts to attack/threaten people.
Aggressive to panda	Reacts in a hostile way or attempts to attack/threaten other pandas.
Friendly to people	Initiates proximity reacts socially to people.
Friendly to panda	Initiates proximity reacts socially to other pandas.
Cooperative	Actively participates in behavioral training and cooperates to complete training tasks.
Olfactory Investigation	Sniffs for a long time at scent-marked locations.
Active	Activity amount (e.g., walking, running, climbing, defecating, scent marking).
Utilization of enrichment	Duration of animal–enrichment interactions.
Playful	Playing duration (e.g., gamboling, rolling or using objects for entertainment).
Forage	Duration of foraging, including processing or consuming food.
Alert	Pays attention to surroundings and changes in surroundings.
Sensitive	Overreacts to minor disturbances in objects or situations.
Anxious	Easily disturbed or frightened, often in a state of restlessness.
Fearful	Retreats readily from people or pandas.
Adverse reactions to noise	Exhibits adverse reactions to anthropogenic noise (e.g., escaping, pacing, reducing foraging).

**Table 4:** Factor loadings of personality traits in the keeper assessment survey.

Personality Traits	PC 1	PC 2
Active	0.906	0.192
Utilization of enrichment	0.880	0.192
Forage	0.876	−0.119
Adverse reactions to noise	−0.109	0.925
Sensitive	0.158	0.884
Alert	0.512	0.638
Eigenvalue	3.058	1.737
Percent of variance	44.380%	35.538%
Cronbach’s alpha	0.865	0.792

## Data Availability

The original contributions presented in this study are included in the article. Further inquiries can be directed to the corresponding author.

## Abbreviations

The following abbreviations are used in this manuscript:

AFT	Accelerated Failure Time model
CI	Confidence Interval
Cr	Creatinine
CRBGPB	Chengdu Research Base of Giant Panda Breeding
ELISA	Enzyme-Linked Immunosorbent Assay
HPA axis	Hypothalamic–Pituitary–Adrenal axis
HR	Hazard Ratio
HRP	Horseradish Peroxidase
ICC	Intraclass Correlation Coefficient
IgG	Immunoglobulin G
KMO	Kaiser–Meyer–Olkin statistic
KM	Kaplan–Meier
PCA	Principal Component Analysis
PC	Principal Component
TR	Time Ratio
